# Treatment of Internet Addiction with Anxiety Disorders: Treatment Protocol and Preliminary Before-After Results Involving Pharmacotherapy and Modified Cognitive Behavioral Therapy

**DOI:** 10.2196/resprot.5278

**Published:** 2016-03-22

**Authors:** Veruska Andrea Santos, Rafael Freire, Morená Zugliani, Patricia Cirillo, Hugo Henrique Santos, Antonio Egidio Nardi, Anna Lucia King

**Affiliations:** ^1^ Laboratory of Panic and Respiration, Institute of Psychiatry of The Federal University of Rio de Janeiro (IPUB/UFRJ) Federal University of Rio de Janeiro (UFRJ) Rio de Janeiro Brazil; ^2^ Institute of Mathematics and Statistics Department of Statistics Fluminense Federal University (UFF) Rio de Janeiro Brazil

**Keywords:** Internet Addiction, Panic Disorder, Generalized Anxiety Disorder, Treatment, Cognitive Behavioral Therapy, Anxiety Disorders, Cognitive Therapy, Therapeutics

## Abstract

**Background:**

The growth of the Internet has led to significant change and has become an integral part of modern life. It has made life easier and provided innumerous benefits; however, excessive use has brought about the potential for addiction, leading to severe impairments in social, academic, financial, psychological, and work domains. Individuals addicted to the Internet usually have comorbid psychiatric disorders. Panic disorder (PD) and generalized anxiety disorder (GAD) are prevalent mental disorders, involving a great deal of damage in the patient’s life.

**Objective:**

This open trial study describes a treatment protocol among 39 patients with anxiety disorders and Internet addiction (IA) involving pharmacotherapy and modified cognitive behavioral therapy (CBT).

**Methods:**

Of the 39 patients, 25 were diagnosed with PD and 14 with GAD, in addition to Internet addiction. At screening, patients responded to the MINI 5.0, Hamilton Anxiety Rating Scale, Hamilton Depression Rating Scale, Clinical Global Impressions Scale, and the Young Internet Addiction Scale. At that time, IA was observed taking into consideration the IAT scale (cutoff score above 50), while anxiety disorders were diagnosed by a psychiatrist. Patients were forwarded for pharmacotherapy and a modified CBT protocol. Psychotherapy was conducted individually, once a week, over a period of 10 weeks, and results suggest that the treatment was effective for anxiety and Internet addiction.

**Results:**

Before treatment, anxiety levels suggested severe anxiety, with an average score of 34.26 (SD 6.13); however, after treatment the mean score was 15.03 (SD 3.88) (*P*<.001). A significant improvement in mean Internet addiction scores was observed, from 67.67 (SD 7.69) before treatment, showing problematic internet use, to 37.56 (SD 9.32) after treatment (*P*<.001), indicating medium Internet use. With respect to the relationship between IA and anxiety, the correlation between scores was .724.

**Conclusions:**

This study is the first research into IA treatment of a Brazilian population. The improvement was remarkable due to the complete engagement of patients in therapy, which contributed to the success of the treatment from a behavioral perspective, and gave patients the confidence to continue to manage Internet use in their lives.

## Introduction

### Background

The rapid expansion of the Internet and its integration into modern life has led to far-reaching changes in our day-to-day existence. The Internet can provide considerable benefits; however, excessive use has brought about the potential for addiction and caused impairments in social, academic, financial, psychological, and work domains. Internet addiction (IA) is defined as the lack of ability to control Internet use, which causes distress, is time consuming, or results in significant social problems, occupational problems, or financial impairments [1]. Psychological disturbances like loneliness, low self-esteem, poor coping capacity, anxiety, stress, and depression are also present [2-4]. Aggressive behavior can also be related to excessive Internet use [5].

IA is not a recognized disorder in the Diagnostic and Statistical Manual of Mental Disorders (DSM-5) [6], and there is no consensus on diagnosis criteria; however, some researchers suggest features such as salience, mood modification, tolerance, withdrawal, conflict, and relapse, arguing that addictions share elements of biopsychosocial processes [7]. Other often-used diagnosis criteria based on modified criteria for pathological gambling include the following: excessive preoccupation with the Internet; the need to use the Internet for increasing periods of time; unsuccessful efforts to control Internet use; feeling restless, moody, depressed, or irritable when attempting to cut down Internet use; staying online longer than originally intended; loss of a significant relationship, job, or educational opportunity; lying to others to conceal the extent of involvement with the Internet; and using the Internet to escape from problems or to relieve a dysphoric mood. It is considered addiction when 5 or more criteria are present over a 6-month period [8,9].

Given that there are no official diagnosis criteria, researchers have validated several instruments to assess IA, and international prevalence rates vary greatly. The most-used questionnaires are the following: the Young Internet Addiction Test (IAT) [10], the Compulsive Internet Use Scale (CIUS) [11], the Excessive Internet Use Scale (EIU) [12], the Problematic Internet Use Questionnaire (PIUQ) [13], the Chen Internet Addiction Scale (CIAS) [14], The Addiction Profile Index Internet Addiction Form-Screening Version (BAPINT-SV) [15], the Internet Addiction Proneness Scale (KS scale) [16], and Young’s Diagnostic Questionnaire (YDQ) [8]. Accordingly, the worldwide prevalence rates of IA differ greatly and range, approximately, from 1.0% to 18.7% [17].

Anxiety disorders share features of excessive fear and anxiety and related behavioral disturbances. These symptoms cause significant distress in social, occupational, or other areas of functioning. Panic disorder (PD) involves recurrent unexpected panic attacks that are characterized by an abrupt surge of intense fear that reaches a peak in minutes, accompanied by physical and cognitive symptoms like palpitations, sweating, chest pain, fear of losing control, fear of dying, trembling, and nausea. Generalized anxiety disorder (GAD) involves excessive anxiety and worry about daily activities that the patient finds difficult to control and is associated with being easily fatigued, irritability, muscle tension, sleep disturbance, difficulty concentrating, and restlessness [6].

People with multiple dependencies such as alcohol, cigarettes, drugs, food, and sex have a higher risk of developing IA, because they have learned to deal with anxiety and difficulties through compulsive behavior [18]. Individuals with IA usually have comorbid psychiatric disorders and this association aggravates Internet use; the relationship between IA and several psychiatric disorders is significant and has awakened academic interest. Researchers have linked IA with depression [19,20-22], attention deficit and hyperactivity [23-25], generalized anxiety disorder and social anxiety disorder [23,26-28], dysthymia [26], alcohol use disorder [29], eating disorder [30], obsessive compulsive personality disorder, borderline personality disorder and avoidant personality disorder [26], and insomnia [31]. Some researchers have suggested that IA could be a symptom of another diagnosis such as anxiety or depression and not a separate disorder [4,32], and have likened IA to impulse control disorder [2,33-35]; however, others have argued that IA should be diagnosed as a primary disorder [10,36].

These comorbidities play an important role in the treatment of IA, which should emphasize the psychiatric condition and treat abusive Internet use [19]. Studies highlight that IA causes damage in social, physical, and mental aspects of life, generating job loss, divorce, family disagreements, social isolation, academic failure, abandonment or expulsion from school [37,38], insomnia, musculoskeletal pain, tension headaches, malnutrition, fatigue, and blurred vision [31] and cognitive impairments like inattention, difficulty concentrating, procrastination, and incomplete tasks [39,40].

### Treatments

Some pharmacological [41,42] and psychotherapeutic [4,18,43-46] treatments have been proposed and recommended for IA both separately and together [47]. Substantial addiction and IA can share the same neurobiological mechanism, so in this sense, addictive behavior medications can help other dependencies [3]. Medications such as escitalopram [48], citalopram [49], bupropion [41,50], olanzapine [51], quetiapine[52], naltrexone [53], methylphenidate [54], and memantine [55] have all been used to treat IA.

Cognitive-behavioral therapy (CBT) has been shown to be effective in treating IA and has been suggested in many studies [18,43,56-58]. CBT highlights the relationship between thoughts, emotions, and behaviors, and teaches patients to pay attention to these and to be ready to identify addictive behavior triggers through their thoughts and feelings. CBT psychotherapists teach coping styles, and promote adherence to treatment, changing behaviors, and preventing relapses [58]. As a treatment for IA, some researchers have suggested traditional CBT [43,44,59-62], CBT and counseling [63], CBT with electroacupuncture (EA) [64,65], CBT and motivational interviewing (MI) [66], CBT and medication [59,61,67], cognitive or behavioral therapy [68], and a modified CBT program titled short-term treatment of Internet and computer addiction (STICA) with individual and group interventions [44].

Group psychotherapy and hospitalization for detoxification are also models of treatment for IA [5]; in addition, multimodal approaches using CBT, psychotherapy with families, treatment of comorbidities, medications, and hospitalization are also suggested [69].

Therefore, the main objective of this study is to test the efficacy of a treatment for PD or GAD and IA involving pharmacotherapy and modified CBT. A secondary aim is to produce clinical research data to corroborate the recognition of IA as a behavioral addiction and ascertain the nature of the relationship between anxiety disorders and IA.

## Methods

The inclusion criteria adopted were the following: (1) patients between 18 and 65 years of age with IA; (2) a diagnosis of PD or GAD through the Mini International Psychiatric Interview (MINI), and confirmed by a psychiatrist; (3) attending and completing the initial interview; and (4) having sufficient cognitive ability to understand the instructions. Patients who did not know how to read or write, or had Axis II pathology [6], were excluded.

This study was approved by the Ethics Committee of The Federal University of Rio de Janeiro, CAAE 2704531460000526. All patients signed a consent form and attended the Laboratory of Panic and Respiration at the Institute of Psychiatry of the Federal University of Rio de Janeiro (IPUB/UFRJ).

All patients were seeking treatment for anxiety symptoms. At screening, they responded to the following scales: MINI 5.0 [70], the Hamilton Anxiety Rating Scale (HAM-A) [71], the Hamilton Depression Rating Scale (HDRS) [72], Clinical Global Impressions Scale (CGI) [73], and the Young Internet Addiction Test (IAT) [10]. IA was assessed through the IAT (scores above 50), while anxiety disorders were diagnosed by a psychiatrist. Patients were then invited to participate in this study and were forwarded for pharmacotherapy and a modified CBT protocol.

Patients were evaluated by a psychiatrist at the beginning of treatment, were permitted to take medication prescribed by the psychiatrist during treatment, and were accompanied by a psychiatrist throughout treatment.

All 39 patients underwent psychotherapy (modified CBT), which was conducted once a week for 10 weeks. The focus was to teach patients how to manage anxiety symptoms without using the Internet, and to promote conscious use of the Internet. Psychotherapy followed 4 phases: psychoeducation about anxiety and Internet use, cognitive reappraisal, behavioral modification, and prevention of relapse (Table 1).

**Table 1:** Description of psychotherapy.

Phase	Description
1 (3 sessions)	Psychoeducation about anxiety (PD or GAD) and Internet use, identifying triggers that increase anxiety and Internet use. Breathing retraining, breathing exercises, and strategies to manage anxiety without using the Internet. Maintenance factors: personal, situational, social, psychiatric, or occupational conditions.
2 (2 sessions)	Cognitive reappraisal of anxiety and Internet use. Daily Internet use and cognitions involving this use and anxiety. “Just a few more minutes on the Internet won’t do me any harm.” “I have to answer my friends immediately, otherwise they will not forgive me.” “If my friends don’t give “likes” on my posts or my photos, it is a signal that they don’t like me or that I did something wrong.” “If I disconnect from the Internet, I will miss important things because the best things are on the Internet.”
3 (3 sessions)	Behavioral modification, breaking routine in the use of the Internet. Training time management with a diary of Internet use, changing ways of dealing with family, friends, social activities, physical exercises, and other aspects of life. Insert positive emotion into daily activities to develop social skills to promote less Internet usage and more in-person interactions.
4 (2 sessions)	Reinforcement of continued recovery and relapse prevention through new beliefs and behaviors, social skills like assertiveness, problem solving, verbal communication, and empathy. Achievement card. Follow-up of scales.

The first phase of psychotherapy lasts 3 sessions and is focused on psychoeducation about the anxiety mechanism, identifying frightening situations, and triggers that increase anxiety and problematic Internet use. The focus is on teaching breathing retraining through breathing exercises and strategies, without using the Internet to deal with anxious thoughts and situations. During this phase, patients learn to identify and accept emotions and to stop fighting against their anxiety. Patients come to understand their anxiety and its relationship to Internet use through self-monitoring of their Internet use during situations involving anxiety. Other maintenance factors related to Internet abuse and anxiety are also explored. These factors can include personal, situational, social, psychiatric, or occupational conditions.

The second phase pertains to cognitive reappraisal of anxiety and Internet use. During this stage, patients think about their daily Internet use, cognitions involved in this use, and anxiety. Cognitive distortions are identified and the patient comes to understand that distortions such as the following contribute to their excessive use of the Internet: “Just a few more minutes on the Internet won’t do me any harm”; “I have to answer my friends immediately, otherwise they will not forgive me”; “If my friends don’t give “likes” on my posts or my photos, it is a signal that they don’t like me or that I did something wrong”; and “If I disconnect from the Internet, I will miss important things because the best things are on the Internet.” All thoughts related to anxiety and Internet use are restructured and new thoughts are proposed; alternative beliefs are generated over 2 sessions.

The third phase (3 sessions) involves behavioral modification with exposure to feared/ansiogenic situations, time management training, and proposal of a diary of Internet use. Behavioral modification involves breaking routines in the use of the Internet, and includes changing ways of dealing with family, friends, social activities, physical exercise, and other aspects of life. All components of situations are analyzed, and replaced or removed as necessary to do things differently and successfully change old ways of functioning. Another important element of this stage is the insertion of positive emotions into daily activities to develop social skills, so as to promote less Internet usage and more in-person interactions. According to positive psychology, enhancing positive emotion increases resilience, helping to reduce signs and symptoms of anxiety and depression and prevent relapse [74].

The fourth phase lasts 2 sessions with a focus on continued recovery and relapse prevention by reinforcing new beliefs and behaviors, and social skills such as assertiveness, problem solving, verbal communication, and empathy. Achievements/improvements are registered on a card (achievement card) and patients are encouraged to continue putting into practice what they have learned in psychotherapy. In the last session, volunteers responded to the same scales used at the start of treatment (IAT, HAM-A, HAM-D, and CGI), to follow up and verify improvements in scale scores. In addition to improvements in scale scores, other important criteria were reduced time spent on the Internet, increased in-person interactions, and in particular, reduced need to use the Internet to escape from problems or manage anxiety.

## Results

This open trial study proposed pharmacological and psychotherapeutic interventions to treat patients diagnosed with PD or GAD and IA. Initially, 41 patients fulfilled criteria and were selected to receive psychotherapy treatment for PD or GAD and IA; however, two did not proceed with treatment (a 33-year-old male taxi driver with PD and IA who moved to another state after the third session; and a 36-year-old female with PD and IA as well as other diagnoses such as an eating disorder and recurrent depression, who attended only 2 sessions of psychotherapy). The other 39 patients attended all sessions; demographic characteristics are presented in Table 2.

Psychiatrists prescribed medications to treat PD or GAD and IA. Some of the medications used were antidepressants such as fluoxetine, sertraline, venlafaxine, desvenlafaxine, paroxetine, escitalopram, zolpidem, and duloxetine; anxiolytics such as clonazepam and alprazolam; psychostimulants such as methylphenidate; and antipsychotics such as quetiapine.

Of the 39 patients, 25 were diagnosed with PD and 14 with GAD, besides also having IA. Before treatment, anxiety levels on the HAM-A suggested severe anxiety, with an average score of 34.26 (SD 6.13); after treatment, the average score was 15.03 (SD 3.88). The IAT average score at the beginning of treatment was 67.67 (SD 7.69), indicating problematic Internet use; after the sessions, the average IAT score was 37.56 (SD 9.32), indicating moderate Internet use and a significate improvement in addiction. The mean HDRS score at baseline was 16.72 (SD 5.56), suggesting mild depression, whereas after treatment the mean score was 7.28 (SD 2.52), indicating no depression. Results of the *t* tests comparing scores before and after treatment are reported in Table 3.

**Table 2:** Sample characteristics.

Characteristic	Mean (SD) or n (%)
Age	28.56 (5.93) (range 19–42)
Female	27 (69%)
Male	12 (31%)
Elementary school	10 (26%)
High school	29 (74%)
Single	28 (72%)
Married	10 (26%)
Widow	1 (3%)
Student/employed	36 (92%)
Unemployed	3 (8%)

**Table 3:** Results of *t* -tests comparing scores before and after treatment.

	Mean (SD)		*t* -test	
	Baseline	Final	*t*	*P* value
IAT^a^	67.67 (7.69)	37.56 (9.32)	13.61	< .001
HDRS^b^	16.72 (5.56)	7.28 (2.52)	8.94	< .001
HAM-A^c^	34.26 (6.13)	15.03 (3.88)	13.62	< .001
CGI^d^	5.15 (0.65)	1.10 (0.24)	27.62	< .001

^a^IAT: Internet Addiction Test

^b^HDRS: Hamilton Depression Rating Scales

^c^HAM-A: Hamilton Anxiety Rating Scale

^d^CGI: Clinical Global Impressions Scale

Correlations between scale scores were also computed. The correlation between scores on the IAT and HAM-A was .724, between scores on the HAM-A and HDRS was .815, and between scores on the IAT and HDRS was .535.

At the end of psychotherapy, all patients felt very positive about their treatment and were very confident, after having recovered their social lives. Patients showed improvements in anxiety symptoms and managing anxiety without use of the Internet. Internet use after treatment became conscious and all patients were classified as mild users. These achievements show that patients were able to recover healthy functioning.

## Discussion

In the present study, the authors described a protocol for modified CBT treatment, examined the effects of this treatment and pharmacotherapy on 39 patients with PD/GAD and IA, and analyzed the relationship between anxiety and IA. Despite controversy regarding the recognition of IA as an official disorder, the harmful effects of this behavioral addiction are highlighted in several studies [75-80]. The psychotherapy protocol was shown to be effective in the treatment of anxiety and IA, since all patients learned to manage anxiety without the Internet and showed conscious use at the end of the sessions.

Several studies have confirmed the association between depression and IA [19-23,27]; however, few studies have explored the association between anxiety and IA [26,81,82]. Imaging studies indicate that IA functions similarly to impulse control disorder; magnetic resonance imaging has shown that the areas activated when an individual with IA has the urge to use the Internet are the same areas activated by addictive substances [5]. At the same time, anxiety plays an important role in increasing Internet usage and strengthening the addiction. The authors highlighted the relationship between anxiety disorders and IA through the correlation shown (.724), which reflects the fact that beliefs and behaviors related to anxiety have an important impact on Internet use and contact with the world.

Previous treatments for IA have been described in the literature such as CBT [45,56,60,83], CBT and medication [59,67,68], and multimodal programs involving individual and group therapy, counselling, and family therapy [44,61,84].

A limitation of the study was the small sample size (39 participants); however, results showed the effectiveness of the proposed treatment, both in reducing symptoms of anxiety and promoting healthy Internet use, to improve IA in patients. Furthermore, this study is the first published research on IA treatment in a Brazilian population.

Future research should identify possible treatments for IA using new strategies and approaches, such as gestalt, counselling, family therapy, mindfulness, psychodynamic therapies, positive psychology, and transdiagnostic treatment. Investigation and analysis should also be conducted to develop new treatments for specific populations in which IA has a harmful impact, such as couples with marital problems, individuals who suffer from insomnia, individuals with attention deficit disorder, and individuals with other addictive behaviors, such as smoking, drug use, eating, sex, or shopping.

Our findings suggest that pharmacotherapy and the developed protocol of psychotherapy in the treatment of patients with anxiety and IA were effective strategies. Improvement was remarkable due to complete engagement of patients in therapy, which contributed to the success of treatment from a behavioral perspective, and gave patients confidence to continue and to manage Internet use in their lives.

IA is increasing around the world and in some countries, such as South Korea and China, it is considered a public health condition. In this sense, effective treatments should be proposed and reported that promote conscious use of the Internet, and involve valuing family, friends, a social life, and physical exercise. As such, Internet use should be conscious so as not to become abuse, and interaction over the Internet should reinforce and expand in-person interactions.

## Abbreviations

BAPINT-SV: Addiction Profile Index Internet Addiction Form-Screening Version
CBT: cognitive-behavior therapy
CGI: Clinical Global Impressions Scale
CIAS: Chen Internet Addiction Scale
CIUS: Compulsive Internet Use Scale
DSM: Diagnostic and Statistical Manual of Mental Disorders
EA: electroacupuncture
EIU: Excessive Internet Use Scale
GAD: generalized anxiety disorder
HAM-A: Hamilton Anxiety Rating Scale
HDRS: Hamilton Depression Rating Scale
IA: internet addiction
IAT: Internet Addiction Test
IPUB/UFRJ: Institute of Psychiatry of the Federal University of Rio de Janeiro
MI: motivational interviewing
MINI: Mini International Psychiatric Interview
PD: panic disorder
PIUQ: Problematic Internet Use Questionnaire
STICA: short-term treatment of Internet and computer addiction
YDQ: Young’s Diagnostic Questionnaire

## References

[1] Shapira NA, Goldsmith TD, Keck PE, Khosla UM, McElroy SL (2000). Psychiatric features of individuals with problematic internet use. J Affect Disord.

[2] Beard KW, Wolf EM (2001). Modification in the proposed diagnostic criteria for Internet addiction. Cyberpsychol Behav.

[3] Brezing C, Derevensky JL, Potenza MN (2010). Non-substance-addictive behaviors in youth: Pathological gambling and problematic Internet use. Child Adolesc Psychiatr Clin N Am.

[4] Cash H, Rae CD, Steel AH, Winkler A (2012). Internet addiction: A brief summary of research and practice. Curr Psychiatry Rev.

[5] Yen Cheng-Fang, Yen J, Ko C (2010). Internet addiction: Ongoing research in Asia. World Psychiatry.

[6] American Psychiatric Association (2013). Diagnostic and Statistical Manual of Mental Disorders, 5th edition (DSM-5).

[7] Griffiths MD (2005). A “components” model of addiction within a biopsychosocial framework. J Subst Use.

[8] Young K (1996). Internet addiction: The emergence of a new clinical disorder. http://chabad4israel.org/tznius4israel/newdisorder.pdf.

[9] Young KS (2004). Internet addiction: A new clinical phenomenon and its consequences. Am Behav Sci.

[10] Young K (1998). Caught in the Net: How to Recognize the Signs of Internet Addiction - And a Winning Strategy for Recovering.

[11] Meerkerk G, Van Den Eijnden RJ, Vermulst AA, Garretsen HF (2009). The Compulsive Internet Use Scale (CIUS): Some psychometric properties. Cyberpsychol Behav.

[12] Johansson A, Götestam KG (2004). Internet addiction: Characteristics of a questionnaire and prevalence in Norwegian youth (12-18 years). Scand J Psychol.

[13] Demetrovics Z, Szeredi B, Rózsa S (2008). The three-factor model of Internet addiction: The development of the Problematic Internet Use Questionnaire. Behav Res Methods.

[14] Chen SH, Weng LC, Su YJ, Wu HM, Yang PF (2003). Development of Chinese internet addiction scale and its psychometric study. Chin J Psychol.

[15] Ogel K, Karadag F, Satgan D (2012). Psychometric properties of the Addiction Profile Index Internet Addiction Form (BAPINT). Bulletin of Clinical Psychopharmacology.

[16] Kim DI, Chung YJ, Lee EA, Kim DM, Cho YM (2008). Development of Internet Addiction Proneness Scale-Short Form (KS scale). Korean J Couns.

[17] Pontes H, Kuss D, Griffiths MD (2015). Clinical psychology of Internet addiction: A review of its conceptualization, prevalence, neuronal processes, and implications for treatment. Neurosci Neuroecon.

[18] Young K (2011). CBT-IA: The first treatment model for Internet addiction. J Cogn Psychother.

[19] Young K, Rogers R (1998). The relationship between depression and Internet addiction. Cyberpsychol Behav.

[20] Ha JH, Kim SY, Bae SC, Bae S, Kim H, Sim M, Lyoo IK, Cho SC (2007). Depression and Internet addiction in adolescents. Psychopathol.

[21] Morrison CM, Gore H (2010). The relationship between excessive Internet use and depression: A questionnaire-based study of 1,319 young people and adults. Psychopathol.

[22] Orsal O, Orsal O, Unsal A, Ozalp SS (2013). Evaluation of Internet addiction and depression among university students. Procedia Soc Behav Sci.

[23] Yen JY, Ko CH, Yen CF, Wu HY, Yang MJ (2007). The comorbid psychiatric symptoms of Internet addiction: Attention deficit and hyperactivity disorder (ADHD), depression, social phobia, and hostility. J Adolesc Health.

[24] Yen J, Yen C, Chen C, Tang T, Ko C (2009). The association between adult ADHD symptoms and internet addiction among college students: The gender difference. Cyberpsychol Behav.

[25] Yen C, Chou W, Liu T, Yang P, Hu H (2014). The association of Internet addiction symptoms with anxiety, depression and self-esteem among adolescents with attention-deficit/hyperactivity disorder. Compr Psychiatry.

[26] Bernardi S, Pallanti S (2009). Internet addiction: A descriptive clinical study focusing on comorbidities and dissociative symptoms. Compr Psychiatry.

[27] Carli V, Durkee T, Wasserman D, Hadlaczky G, Despalins R, Kramarz E, Wasserman C, Sarchiapone M, Hoven CW, Brunner R, Kaess M (2013). The association between pathological internet use and comorbid psychopathology: A systematic review. Psychopathol.

[28] Adalier A (2012). The relationship between Internet addiction and psychological symptoms. Int J Glob Educ.

[29] Ko C, Yen J, Chen C, Chen S, Wu K, Yen C (2006). Tridimensional personality of adolescents with internet addiction and substance use experience. Can J Psychiatry.

[30] Tao ZL, Liu Y (2009). Is there a relationship between Internet dependence and eating disorders? A comparison study of Internet dependents and non-Internet dependents. Eat Weight Disord.

[31] Cheung LM, Wong WS (2011). The effects of insomnia and internet addiction on depression in Hong Kong Chinese adolescents: An exploratory cross-sectional analysis. J Sleep Res.

[32] Kratzer Silvia, Hegerl U (2008). [Is "Internet Addiction" a disorder of its own?--a study on subjects with excessive internet use]. Psychiatr Prax.

[33] Shapira NA, Lessig MC, Goldsmith TD, Szabo ST, Lazoritz M, Gold MS, Stein DJ (2003). Problematic internet use: Proposed classification and diagnostic criteria. Depress Anxiety.

[34] Aboujaoude E, Koran LM, Gamel N, Large MD, Serpe RT (2006). Potential markers for problematic internet use: A telephone survey of 2,513 adults. CNS Spectr.

[35] Block JJ (2008). Issues for DSM-V: Internet addiction. Am J Psychiatry.

[36] Pies R (2009). Should DSM-V designate "Internet addiction" a mental disorder?. Psychiatry (Edgmont).

[37] Mythily S, Qiu S, Winslow M (2008). Prevalence and correlates of excessive Internet use among youth in Singapore. Ann Acad Med Singapore.

[38] Wang L, Luo J, Luo J, Gao W, Kong J (2012). The effect of Internet use on adolescents' lifestyles: A national survey. Comput Human Behav.

[39] Davis R (2001). A cognitive-behavioral model of pathological Internet use. Comput Human Behav.

[40] Kaneez FS, Zhu K, Tie L, Osman NBH (2013). Is cognitive behavioral therapy an intervention for possible Internet addiction disorder?. J Drug Alcohol Res.

[41] Han DH, Renshaw PF (2012). Bupropion in the treatment of problematic online game play in patients with major depressive disorder. J Psychopharmacol.

[42] Paik A, Oh D, Kim D (2014). A case of withdrawal psychosis from internet addiction disorder. Psychiatry Investig.

[43] Young K (2009). Internet addiction: Diagnosis and treatment considerations. J Contemp Psychother.

[44] Jäger S, Müller KW, Ruckes C, Wittig T, Batra A, Musalek M, Mann K, Wölfling K, Beutel ME (2012). Effects of a manualized short-term treatment of internet and computer game addiction (STICA): Study protocol for a randomized controlled trial. Trials.

[45] Young KS (2013). Treatment outcomes using CBT-IA with Internet-addicted patients. J Behav Addict.

[46] King ALS, Nardi AE, Cardoso AS (2014). Nomofobia: Dependência do Computador, Internet, Redes Sociais? Dependência do Telefone Celular?.

[47] Przepiorka AM, Blachnio A, Miziak B, Czuczwar SJ (2014). Clinical approaches to treatment of Internet addiction. Pharmacol Rep.

[48] Dell'Osso B, Hadley S, Allen A, Baker B, Chaplin W, Hollander E (2008). Escitalopram in the treatment of impulsive-compulsive internet usage disorder: An open-label trial followed by a double-blind discontinuation phase. J Clin Psychiatry.

[49] Sattar P, Ramaswamy S (2004). Internet gaming addiction. Can J Psychiatry.

[50] Han DH, Hwang JW, Renshaw PF (2010). Bupropion sustained release treatment decreases craving for video games and cue-induced brain activity in patients with Internet video game addiction. Exp Clin Psychopharmacol.

[51] McElroy SL, Nelson E, Welge J, Kaehler L, Keck P (2008). Olanzapine in the treatment of pathological gambling: A negative randomized placebo-controlled trial. J Clin Psychiatry.

[52] Atmaca M (2007). A case of problematic internet use successfully treated with an SSRI-antipsychotic combination. Prog Neuropsychopharmacol Biol Psychiatry.

[53] Bostwick JM, Bucci JA (2008). Internet sex addiction treated with naltrexone. Mayo Clin Proc.

[54] Han DH, Lee YS, Na C, Ahn JY, Chung US, Daniels MA, Haws CA, Renshaw PF (2009). The effect of methylphenidate on Internet video game play in children with attention-deficit/hyperactivity disorder. Compr Psychiatry.

[55] Camardese G, DeRisio L, DiNicola M, Pizi G, Janiri L (2012). A role for pharmacotherapy in the treatment of "internet addiction". Clin Neuropharmacol.

[56] Hall A, Parsons J (2001). Internet addiction: College student case study using best practices in behavior therapy. J Ment Health Couns.

[57] Sato T (2006). Internet addiction among students: Prevalence and psychological problems in Japan. Japan Med Assoc J.

[58] King DL, Delfabbro PH, Griffiths MD, Gradisar M (2011). Assessing clinical trials of Internet addiction treatment: A systematic review and CONSORT evaluation. Clin Psychol Rev.

[59] Kaneez FS, Zhu K, Tie L, Osman NBH (2013). Is cognitive behavioral therapy an intervention for possible Internet addiction disorder?. J Drug Alcohol Res.

[60] Ge L, Ge X, Xu Y, Zhang K, Zhao J, Kong X (2011). P300 change and cognitive behavioral therapy in subjects with internet addiction disorder: A 3-month follow-up study. Neural Regen Res.

[61] Wölfling K, Müller K, Beutel M (2012). AS32-02 - Treating internet addiction: First results on efficacy of a standardized cognitive-behavioral therapeutic approach. Eur Psychiatry.

[62] Li H, Wang S (2013). The role of cognitive distortion in online game addiction among Chinese adolescents. Chid Youth Serv Rev.

[63] Fang-ru Y, Wei H (2005). The effect of integrated psychosocial intervention on 52 adolescents with internet addiction disorder. Chinese J Clin Psychol.

[64] Zhu T, Jin R, Zhong X (2009). [Clinical effect of electroacupuncture combined with psychologic interference on patient with Internet addiction disorder]. Zhongguo Zhong Xi Yi Jie He Za Zhi.

[65] Zhu T, Li H, Jin R, Zheng Z, Luo Y, Ye H, Zhu H (2012). Effects of electroacupuncture combined psycho-intervention on cognitive function and event-related potentials P300 and mismatch negativity in patients with internet addiction. Chin J Integr Med.

[66] van Rooij AJ, Zinn MF, Schoenmakers TM, van de Mheen D (2010). Treating internet addiction with cognitive-behavioral therapy: A thematic analysis of the experiences of therapists. Int J Ment Health Addiction.

[67] Santos V, Nardi A, King A (2015). Treatment of internet addiction in patients with panic disorder and obsessive compulsive disorder: A case report. CNS Neurol Disord Drug Targets.

[68] Kim S, Han D, Lee Y, Renshaw P (2012). Combined cognitive behavioral therapy and bupropion for the treatment of problematic on line game play in adolescents with major depressive disorder. Computer Human Behav.

[69] Dowling NA, Brown M (2010). Commonalities in the psychological factors associated with problem gambling and Internet dependence. Cyberpsychol Behav Soc Netw.

[70] Sheehan DV, Lecrubier Y, Sheehan KH, Amorim P, Janavs J, Weiller E, Hergueta T, Baker R, Dunbar GC (1998). The Mini-International Neuropsychiatric Interview (M.I.N.I.): The development and validation of a structured diagnostic psychiatric interview for DSM-IV and ICD-10. J Clin Psychiatry.

[71] Hamilton M (1959). The assessment of anxiety states by rating. Br J Med Psychol.

[72] Hamilton M (1960). A rating scale for depression. J Neurol Neurosurg Psychiatry.

[73] Guy W (1976). U.S. Department of Health, Education, and Welfare, Public Health Service, Alcohol, Drug Abuse and Mental Health Administration, NIMH Psychopharmacology Research Branch, Division of Extramural Research Programs.

[74] Wood AM, Tarrier N (2010). Positive clinical psychology: A new vision and strategy for integrated research and practice. Clin Psychol Rev.

[75] Jap T, Tiatri S, Jaya ES, Suteja MS (2013). The development of an Indonesian online game addiction questionnaire. PLoS One.

[76] Ko CH, Yen J, Chen C, Chen C, Yen C (2008). Psychiatric comorbidity of internet addiction in college students: An interview study. CNS Spectr.

[77] Muñoz-Rivas MJ, Fernández L, Gámez-Guadix M (2010). Analysis of the indicators of pathological Internet use in Spanish university students. Span J Psychol.

[78] Park JW, Park K, Lee I, Kwon M, Kim D (2012). Standardization study of internet addiction improvement motivation scale. Psychiatry Investig.

[79] Shek DT, Tang VM, Lo C (2009). Evaluation of an Internet addiction treatment program for Chinese adolescents in Hong Kong. Adolescence.

[80] Sun P, Johnson C, Palmer P, Arpawong T, Unger J, Xie B, Rohrbach L, Spruit-Metz D, Sussman S (2012). Concurrent and predictive relationships between compulsive internet use and substance use: Findings from vocational high school students in China and the USA. Int J Environ Res Public Health.

[81] Pezoa-Jares RE, Espinoza-Luna IL, Vasquez-Medina JA (2012). Internet addiction: A review. J Addict Res Ther.

[82] Berner J, Santander J, Contreras A, Gómez T (2014). Description of internet addiction among Chilean medical students: A cross-sectional study. Academic Psychiatry.

[83] Du Y, Jiang W, Vance A (2010). Longer term effect of randomized, controlled group cognitive behavioural therapy for Internet addiction in adolescent students in Shanghai. Aust N Z J Psychiatry.

[84] Goldsmith T, Shapira N, Hollander E, Stein DJ (2006). Problematic internet use. Clinical Manual of Impulse-Control Disorders.

